# Ninth International Congress of Hygiene and Demography

**Published:** 1898-02

**Authors:** 


### NINTH INTERNATIONAL CONGRESS OF HYGIENE AND DEMOGRAPHY.

### VETERINARY HYGIENE—CIVIL AND MILITARY.

Papers: 1. Means for the prevention of the spread of tuberculosis in domestic animals and the transmission of the same to the human species.

2. Necessity and advisability of sanitary regulations for domestic animals, on the point of view of their diseases, and of the consumption of their flesh and alimentary substances.

3. Conditions which ought to be required in beasts destined to the obtension of serums and vaccines, and intervention which is to be assigned to the veterinary surgeon.

4. Hygienic and prophylactic measures for the prevention of glanders in military live-stock.

5. Veterinary, hygienic, and sanitary prescriptions in cavalry barracks.

6. Preventive antitetanic-serum in military live-stock, with the advantages derived from its employment.

